# Molecular characterization of normal and physiologically suppressed human parathyroid glands at single-cell resolution

**DOI:** 10.1210/jendso/bvag133

**Published:** 2026-06-23

**Authors:** Timofey Glinin, Sergey Isaev, Anastasia Shubina, Igor Adameyko, Elham Khanafshar, Julie A Sosa, James Koh

**Affiliations:** Laboratory of Endocrine Neoplasia, Department of Surgery, University of California at SanFrancisco, San Francisco, CA 94143, USA; Department of Neuroimmunology, Center for Brain Research, Medical University Vienna, Vienna 1090, Austria; Open Longevity, Los Angeles, CA 91403,USA; Department of Neuroimmunology, Center for Brain Research, Medical University Vienna, Vienna 1090, Austria; Department of Physiology and Pharmacology, Karolinska Institute, Solna 17165, Sweden; Department of Pathology, University of California at SanFrancisco, San Francisco, CA 94143, USA; Laboratory of Endocrine Neoplasia, Department of Surgery, University of California at SanFrancisco, San Francisco, CA 94143, USA; Department of Surgery, University of California at SanFrancisco, San Francisco, CA 94143, USA; Laboratory of Endocrine Neoplasia, Department of Surgery, University of California at SanFrancisco, San Francisco, CA 94143, USA

**Keywords:** parathyroid, hyperparathyroidism, single nuclei sequencing, primary hyperparathyroidism

## Abstract

Primary hyperparathyroidism (PHPT) is a common endocrine neoplastic disorder caused by excessive parathyroid hormone (PTH) secretion, predominantly due to single gland adenomas that arise in 1 of the 4 parathyroid glands. In the presence of a dominant hypersecretory adenoma, the remaining normal glands become functionally suppressed with attenuated PTH output. The mechanisms underlying suppression remain poorly understood. We performed single-nucleus RNA sequencing combined with immunofluorescence analysis to compare normal and suppressed human parathyroid glands. Transcriptomic analysis of 1.7 billion reads from 83 693 nuclei revealed 4 distinct parathyroid cell populations, including a PDE10A^+^ subtype markedly enriched in suppressed glands and a REXO5^+^ subtype present only in unsuppressed tissue. In suppressed glands, the scavenger receptor LRP2 (Megalin) was strongly up-regulated. Ex vivo treatment of parathyroid glands with an inactivating LRP2 antibody increased PTH secretion, consistent with relief of physiological suppression. Suppressed parathyroid glands internalized extracellular, fluorescently labeled synthetic PTH, an activity blocked by a specific LRP2 antagonist. In parallel, suppression coincided with an increase in PDE10A+ cells and a concomitant reduction in cells expressing PDE11A. PDE11 and PDE10 inhibition selectively enhanced PTH secretion in normal and suppressed glands, respectively. These data suggest that physiological suppression of PTH secretion in normal tissue may be achieved through extracellular PTH scavenging in concert with modulation of intracellular signaling via PDE isoform switching.

Human parathyroid glands maintain serum calcium homeostasis through the regulated, calcium-responsive secretion of the parathyroid hormone (PTH), which elicits responses in multiple downstream tissues to control circulating levels of this essential mineral. Disorders that perturb this dynamic and finely balanced feedback mechanism are common: primary hyperparathyroidism (PHPT), caused by parathyroid adenomas that secrete inappropriately elevated amounts of PTH, is diagnosed in about 100 000 adults in the United States each year [1-3]. Despite this significant clinical burden, relatively little is known at the molecular level about the cellular composition of normal human parathyroid glands. Cellular subtyping within the parathyroid has historically been limited to histomorphological descriptors focusing on parathyroid tumors, with chief cells identified as the predominant cell type responsible for gland function and oxyphil cells as a secondary, less abundant component of unknown significance [4, 5]. In parathyroid adenomas, our group and others have reported differences in molecular etiology and mutational profiles [6-8], calcium responsiveness among parathyroid tumor cells [9, 10], and heterogeneity in cellular and molecular profile alterations in response to vitamin D deficiency [11]. These findings suggest that the normal parathyroid gland is not a monolithic entity and that functionally significant differences exist in the responsiveness of currently unrecognized, discrete parathyroid cell types to physiological stressors and neoplastic transformation.

To document the cellular composition and transcriptional profiles of normal human parathyroid tissue, we performed a single-nuclei RNAseq (snRNAseq) study of 6 live tissue cadaveric parathyroid glands from 4 individual donors. During procurement, one of the presumptive normal donors was found to have a single grossly enlarged, hypercellular parathyroid gland, elevated serum PTH (140 pg/mL) and hypercalcemia (11.2 mg/dL), consistent with PHPT. In patients with a solitary parathyroid adenoma, the predominant presentation in PHPT [12], PTH secretion in the remaining normal parathyroid glands is functionally suppressed by the dominant tumor [13]. To gain insight into the mechanisms natively employed by normal parathyroid glands to suppress PTH secretion, we took advantage of access to live, normal tissue from this donor, as normal non-neoplastic glands would not typically be resected during parathyroidectomy for PHPT. We compared the transcriptional profiles of histologically normal suppressed and nonsuppressed parathyroid glands and validated candidate differential gene expression patterns in an independent panel of archival normal and cryopreserved tumor-adjacent histologically normal suppressed parathyroid tissue. This approach allowed us to identify cell-type-specific molecular signatures associated with endogenous PTH down-regulatory behavior. Unsupervised uniform manifold approximation and projection (UMAP) plots revealed the presence of 4 discrete parathyroid populations among the cohort of cells expressing the canonical parathyroid markers PTH and GCM2. Physiological suppression was associated with a marked shift in cellular composition when comparing suppressed vs nonsuppressed normal glands. Pathway analysis of the suppressed gland signature suggested 2 potentially novel mechanisms of PTH down-regulation. Interference with these pathways using an LRP2 inactivating antibody, an LRP2 antagonist, and a pharmacological PDE inhibitor relieved PTH suppression in live tissue ex vivo functional interrogation assays, consistent with their role in mediating down-regulation of PTH secretion. These findings suggest that normal parathyroid glands may employ multiple mechanisms to reduce PTH production in response to a PTH hypersecretory physiological environment. Further exploration of these mechanisms could lead to alternative nonsurgical approaches to mitigate hyperparathyroidism through activation of native suppressive pathways.

## Materials and methods

### Donor sample collection

Normal human parathyroid and thyroid tissue was obtained from 4 cadaveric donors through our institution's solid organ transplant service, using a fully authorized tissue procurement protocol for the recovery of viable, intact parathyroid and thyroid glands [14]. Superior extension of the standard midline sternotomy incision during multi-organ procurement was utilized for en bloc removal of the thyroid. After transport to the laboratory, a Fluo-Optics Fluobeam camera with near-infrared wavelength illumination and software at the high sensitivity setting were employed to guide ex vivo identification and dissection of intact parathyroid glands. The dissected glands were immediately placed in UW solution on an ice-cold tissue culture plate in a laminar flow tissue culture hood. Tissue pieces were then allocated for single nuclei isolation, fixation in 4% paraformaldehyde for histology, or secretion media (MEM-EBSS-CMF media supplemented with 0.5 mM calcium, 0.5 mM magnesium, 0.2% bovine serum albumin, and 20 mM HEPES [pH 7.4]) for ex vivo calcium response assays.

### Single nuclei preparation

Nuclei from parathyroid and thyroid glands were isolated using a published protocol [15] with minor modifications. Initially, tissue samples were placed into 1 mL of lysis buffer at +4 °C and then processed with a TissueLyser II (Qiagen) for one minute at a frequency of 25 Hz. The lysis buffer was freshly prepared on the day of tissue processing. The lysis buffer composition was formulated in nuclease-free water with the following components: 250 mM sucrose (Sigma), 25 mM KCl, 5 mM MgCl2, 10 mM Tris·Cl (pH 8), 1× Protease Inhibitor (Roche, cat. no. 11873580001), 1 μM DTT (Sigma), 0.4 U/μL RNaseIn (Thermo Fisher Scientific, cat. no. AM2684), 0.2 U/μL SuperaseIn (Invitrogen, cat. no. AM2696), and 1% (v/v) Triton X-100. TissueLyser plates were precooled at −20 °C for 15 minutes before use. The samples held on ice for 5 minutes for cell lysis. Following lysis, the homogenized tissue was filtered through a 40-μm cell strainer into a sterile 50-mL conical tube. The filtered samples were centrifuged for 5 minutes at 500 × g at 4 °C to pellet the nuclei. The supernatant was then carefully transferred into a separate 1.5-mL Eppendorf tube and reserved on ice. The nuclear pellet was resuspended in 500 μL of storage buffer (SB), which consisted of 96% PBS, 4% bovine serum albumin (BSA), and 0.2 U/μL Protector RNaseIn (Sigma-Aldrich, cat. no. 03335402001). The suspension was then passed through a 40 micron mesh filter prior to flow cytometry.

To prepare an unstained, negative control, 300 μL of the filtered sample was transferred into a separate FACS sorting tube. Hoechst 33342 stain was then added to the remaining filtered solution, followed by vortexing for 5 seconds and incubation on ice. The samples were held for 15 minutes to stain. Preparative flow cytometry was performed in a BD FACSAria Fusion instrument with a 70-micron nozzle. Flow cytometer gating information is provided in Fig. S2 [16] (Dataset 32209641; https://doi.org/10.6084/m9.figshare.32209641).

Post-FACS, nuclear counts were performed using a hemocytometer and fixed according to the split pool sequencing commercial protocol (Parse Biosciences). The fixed nuclei concentration was between 50 000 to 100 000 per sample. Samples were then frozen at −80 °C prior to beginning the barcoding procedures.

### Barcoding and library preparation

The fixed nuclei samples were retrieved from −80 °C storage, thawed at 37 °C in a water bath, and then placed on ice for counting. The samples were barcoded, and single-nuclei sequencing libraries were prepared via the split-pool method [17] using the Evercode Whole Transcriptome v2 kit (Parse Biosciences). A total of 100 000 nuclei were processed, derived from unsuppressed parathyroid glands from 4 patients (50 000 nuclei), 2 suppressed parathyroid glands from 1 patient (25 000 nuclei), and thyroid glands from 3 patients (25 000 nuclei). Eight libraries were prepared: the first library contained 1000 nuclei to test sequencing conditions, the second library contained 3000 nuclei for deep sequencing validation, and libraries 3 to 8 each contained 16 000 nuclei.

### Single-nuclei RNAseq data analysis

Quality control, filtering, and preprocessing of raw sequencing data were performed using the Trailmaker suite of computational tools (Parse Biosciences) and raw count matrix was preprocessed using the scanpy package [18]. For scRNA-Seq analysis, only cells with more than 1000 expressed genes were retained. Briefly, counts were normalized so that each cell contained a total of 10 000 UMIs, followed by log-transformation and the selection of 3000 highly variable genes. Dimensionality reduction was performed with PCA, and the first 30 principal components were integrated using Harmony [19]. This embedding was used to construct a k-nearest neighbor (kNN, *k* = 20) graph, which was subsequently used for visualization with UMAP [20] and community detection with the Leiden algorithm [21].

Cell type annotation was first performed on the full dataset using clustering at resolution 2. For subsequent analyses restricted to normal parathyroid cells, a new embedding was constructed from the raw counts of parathyroid endocrine cells (donors 1-4), normal parathyroid endocrine cells (donors 1-3), and suppressed parathyroid endocrine cells (donor 4) followed by the same preprocessing steps (using 10 principal components in this case). This ensured that the selection of highly variable genes was tailored to differences among parathyroid subpopulations.

Normal parathyroid endocrine cells were clustered at resolution 1. Clusters with similar expression profiles, as assessed by hierarchical clustering of cluster centroids, were grouped together. Differential expression analyses (for marker gene identification and condition comparisons) were performed on log-transformed counts using a *t*-test with Benjamini–Hochberg FDR correction [22]. Marker genes were defined using thresholds of |log_2_ fold change| ≥ 1.5 and FDR ≤ 1 × 10^−5^. These markers were then used to compute gene signature scores with the “sc.tl.score_genes” function, and the resulting scores were softmax-transformed to generate signature-based embeddings. Scaled expression of selected marker genes across cell type and condition groups was visualized using heatmaps generated from log-normalized expression values. Label transfer between normal and suppressed parathyroid cells was performed using the Symphony algorithm [23]. The integrated Symphony embedding was then used to construct a joint normal/suppressed graph with the batch-balanced kNN algorithm [24].

### Gene ontology enrichment analysis

Gene ontology enrichment analysis was performed by querying the open-source Gene Ontology knowledgebase [25, 26], version 10.5281/zendo.10536401, by using population-specific genes with adjusted *P* value < 1 × 10^−15^ and gene signature score >10. Genesets linked to parathyroid populations were identified by employing GO and AmiGO enrichment tools [27] and a statistical significance threshold of false discovery rate (FDR) <0.04.

### Archival normal glands for immunofluorescence analysis

Previously obtained archived human parathyroid glands with normal histology were used for independent immunofluorescent validation of candidate marker gene expression. Seven donor glands were included for staining analysis. Normal histology was preverified by a board-certified pathologist (E.K.).

### Archived suppressed and adenomatous glands for immunofluorescence and functional analysis

Following IRB approval, a retrospective chart review was conducted to identify cases of patients with primary hyperparathyroidism (PHPT) who underwent parathyroidectomy involving the removal of 2 or more glands, with cryopreserved tissue available for one or more specimens. Cases were included if the pathology report confirmed the presence of at least one hypercellular adenoma and one normocellular gland. Suppression criteria required that the normocellular gland be excised prior to the adenoma and that no marked intraoperative PTH decline occurred following its removal.

Clinical metadata for included cases are summarized in Table 1, detailing the anatomical location of the suppressed gland(s), reason for excision, adenoma location, and surgical removal sequence. Table S1 [16] (Dataset 32209641; https://doi.org/10.6084/m9.figshare.32209641) provides expanded clinical details, including patient sex, age, pre and postoperative serum calcium, PTH, and vitamin D levels, as well as histological findings for each suppressed and adenomatous gland. FFPE sections from suppressed and adenoma glands were subjected to hematoxylin and eosin staining to confirm cellularity. For cases 4 and 10, FFPE blocks of the adenoma were unavailable. Cryopreserved suppressed glands from cases 2 and 10, expired for transplant purposes, were available for experimental use in functional assays.

**Table 1:** Anatomical location and reason for removal of suppressed glands from the validation cohort of PHPT patients

Sample ID	Anatomical location of suppressed gland(s)	Reason for removal	Adenoma location(s)	Removal sequence
2	Right upper	Grossly enlarged in patient with multi-gland disease. ioPTH did not fall after removal and gland was subsequently found to be histologically normocellular.	LU, RL	*LU*—**RU**—*RL*
3	Left upper	Modestly enlarged in patient with multi-gland disease. ioPTH did not fall after removal and gland was subsequently found to be histologically normocellular.	LL, RU	*LL*—**LU**—*RU*
6	Right lower	Ectopic location (cervical thymus); ioPTH did not fall after removal and gland was subsequently found to be histologically normocellular	LU	**RL**—*LU*
9	Left upper	Modestly enlarged in patient with multi-gland disease. ioPTH did not fall after removal and gland was subsequently found to be histologically normocellular.	LL, RU	*LL*-**LU**-*RU*
10	Left lower*^a^*, right upper	Nonlocalizing on preoperative imaging. Grossly enlarged. ioPTH did not fall after removal and glands were subsequently found to be histologically normocellular.	RL (thyrothymic)	**LL—RU**—*RL*
11	Left upper*^a^*, left lower, right lower	Nonlocalizing on preoperative imaging. Grossly enlarged. ioPTH did not fall after removal and suppressed glands were subsequently found to be histologically normocellular.	RU	**LU—LL—RL**—*RU*

Bold = suppressed glands.

*Italics* = gland with culprit adenoma.

Abbreviation: ioPTH, intraoperative PTH.

^*a*^Glands analyzed from the multiple suppressed gland cases.

### Immunofluorescence staining

Formalin-fixed, paraffin-embedded (FFPE) tissue sections of 5 μm thickness were deparaffinized in xylene and rehydrated through a graded series of ethanol concentrations to distilled water. Antigen retrieval was performed by heating the slides in citrate buffer (pH 6.0) using a microwave oven or pressure cooker for 10-20 minutes. After cooling to room temperature, the sections were washed in phosphate-buffered saline with 0.1% Tween-20 (PBST). To block nonspecific binding sites, the sections were incubated with 3% bovine serum albumin in Tris-buffered saline with 0.1% Tween-20 (BSA-TBST) for 30 minutes at room temperature. Primary antibodies specific to the target antigens were applied to the sections and incubated overnight at 4 °C in a humidified chamber. Following primary antibody incubation, the sections were washed 3 times for 5 minutes each in PBST. To visualize the primary antibody, the sections were then incubated for 1 hour at room temperature in the dark with 1:500 dilution of species-appropriate AlexaFluor 488/595/647 labeled secondary antibody (Thermofisher Scientific, Waltham, MA, USA) in BSA-TBST. The sections were then washed again 3 times for 5 minutes each in PBST.

Nuclei were counterstained using a mounting medium containing NucBlue. The stained sections were examined using a Zeiss AxioVert inverted epifluorescence microscope equipped with a multiwavelength LED illuminator and filter sets appropriate for detecting the respective secondary-conjugated fluorophores.

PTH rabbit monoclonal antibodies were used at a 1:250 dilution (custom monoclonal line 38-397-1b, RRID:AB_3750269). IL1R1 mouse monoclonal antibodies were used at 1:500 dilution (Proteintech, 60112-1, RRID:AB_2264785). F13A1 rabbit monoclonal antibodies were used at a 1:500 dilution (Invitrogen, ma5-44662, RRID:AB_2931120). LAMA2 rabbit polyclonal antibodies were used at 1:250 dilution (Antibodies.com, A97434, RRID:AB_3750268). REXO5 rabbit polyclonal antibodies were used at 1:250 dilution (Invitrogen, PA5-59283, RRID:AB_2643420), FKBP5 staining mouse monoclonal antibodies at 1:250 dilution (Origene, TA500673, RRID:AB_2103129), PDE10A mouse monoclonal antibodies at 1:500 dilution (Origene, TA500625, RRID:AB_2162589), PDE11A goat polyclonal antibodies at 1:250 dilution (Invitrogen, PA5-18767, RRID:AB_10986152), PAPPA mouse monoclonal antibodies at 1:500 dilution (Proteintech, 66962-1, RRID:AB_2882285), SORBS1 rabbit polyclonal antibodies 1:250 dilution (Invitrogen, PA5-24183, RRID:AB_2541683). For donor 4 experiments and suppressed gland studies, goat antirabbit polyclonal SORBS1 antibodies at dilution 1:250 (Abcam, AB4551, RRID:AB_304515) and LRP2 rabbit polyclonal antibodies at a dilution of 1:250 (Invitrogen, PA5-92032, RRID:AB_2806358) were used.

### Quantitation of immunofluorescence signals

Immunofluorescence staining was assessed based on the percentage of positive cells and the intensity of the fluorescence signal. Epifluorescence images were obtained using a Zeiss AxioVert A1 microscope (Carl Zeiss) equipped with a Colibri 7 LED illuminator (Carl Zeiss) and a Zeiss AxioCam MRM CMOS camera. Comparisons of images were made between slides imaged using identical imaging acquisition parameters to ensure consistency. All slides were imaged under identical acquisition parameters, with intensity adjustment relative to IgG-control background. Images (2-3 fields per sample at 40× magnification) were exported as TIFF files and analyzed in FIJI software (National Institutes of Health, Bethesda, MD) according to previously described methods [28] with minor modifications. Mean fluorescence intensity (MFI) and Otsu-adjusted thresholded area were quantified automatically, and positive cells were counted manually. Nuclei counts were obtained using the SCF package with the adjusted watershed function, with manual confirmation for randomly selected samples. Statistical analysis was performed using the Mann–Whitney nonparametric test in GraphPad Prism version 8.3.0 (GraphPad Software, San Diego, CA). Data are presented as mean ± SD, and differences were considered significant at *P* ≤ .05.

### Calcium/PTH setpoint ex vivo secretion assays

PTH secretion assays were performed as previously described [14, 29] with minor modifications. Briefly, 5-10 mg of fresh parathyroid tissue or thawed cryopreserved suppressed tissue were placed into Transwell cell culture inserts (polycarbonate membrane, 0.4 µm pore; Corning, Cat# 3413). Inserts were equilibrated for 30 minutes at 37 °C in a 5% CO_2_ incubator, with media on both sides of the Transwell replaced every 15 minutes.

Each Transwell was then sequentially transferred to a new well containing 600 µL of custom-made MEM–EBSS–CMF medium (supplemented with 0.5 mM magnesium, 0.2% bovine serum albumin, and 20 mM HEPES, pH 7.4) at different calcium concentrations (0.5, 0.75, 1.0, 1.25, 1.5, 2.0, and 3.0 mM). At each concentration, tissue was incubated for 15 minutes, with the inner Transwell medium replaced by the current calcium concentration during each transfer. At the end of each incubation step, 350 µL of medium from the outer compartment of each well was collected for PTH analysis.

PTH levels were measured using a commercial ELISA kit (QuidelOrtho, San Diego, USA, Cat. # 60-3000, RRID:AB_2891347) according to the manufacturer's instructions. Each sample was analyzed in at least duplicate, and absorbance was recorded at both 620 and 450 nm using an Absorbance 96 microplate reader (Byonoy, Germany). Setpoint curves and PTHmax were evaluated using Prism software (GraphPad Software, LLC).

For LRP2 inhibition assays, parathyroid tissue fragments were preincubated for 30 minutes with 120 µg/mL of an inactivating LRP2 polyclonal antibody (PA5-92032, ThermoFisher, RRID:AB_2806358) or an equivalent concentration of purified rabbit IgG, prior to equilibration. For PDE11 inhibition assays in normal parathyroid tissue, 10 µM BC11-38 (eMolecules No.: 589794115) was added to both equilibration and setpoint media. For PDE10 inhibition assays in normal parathyroid tissue, 100 nM TP-10 (TargetMol, CAS:898563-00-3) was added to both equilibration and setpoint media.

**Figure 1:**
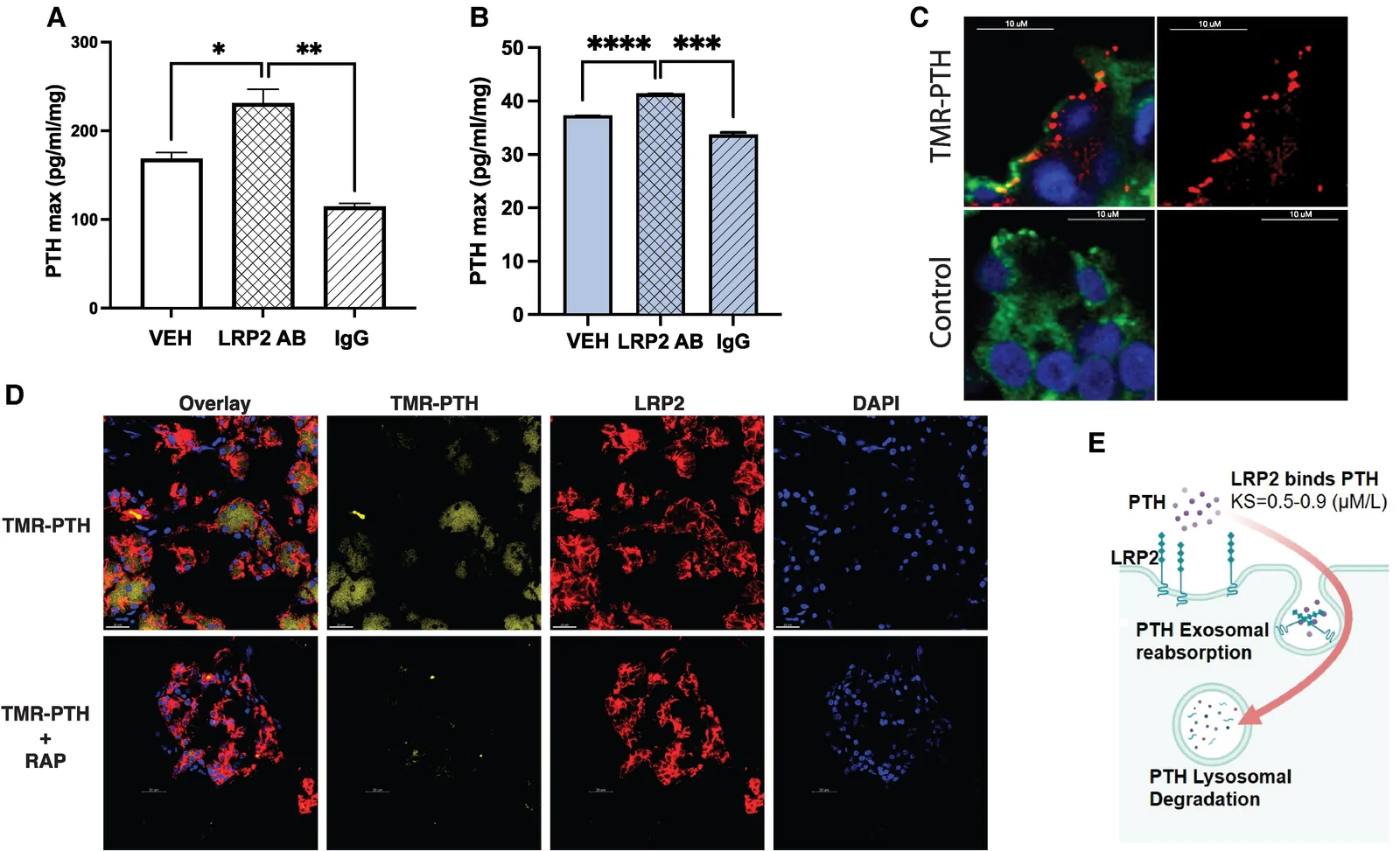
Interference with LRP2 enhances maximal PTH secretion. Quantification of maximal PTH secretion (pg/mL/15 minutes per mg wet weight of tissue) at 0.5 mM extracellular calcium. Bars represent mean ± SD. Statistical significance was determined by one-way ANOVA with post hoc testing; *P < .05, **P < .01, ***P < .001, ****P < .0001. (A) Normal tissue after pretreatment with LRP2-inactivating antibodies (LRP2 AB), control rabbit IgG, or no antibody (NP). (B) Suppressed tissue after pretreatment with LRP2 AB, control IgG, or NP. (C) Confocal imaging of suppressed parathyroid tissue incubated for 30 minutes with TMR-labeled PTH (upper images) or unlabeled PTH (lower images). Blue = Nuclei stained with DAPI Green = plasma membranes stained with 488-WGA. Red = TMR-PTH. Left panels = merged images. Right panels = red channel only. Optical section thickness = 0.5 micron. Scale bar = 10 microns. (D) Confocal images of suppressed parathyroid tissue incubated with TMR-PTH + BSA as a negative control, or with TMR-PTH plus the receptor-associated protein RAP, a specific inhibitor of LRP2 ligand binding. TMR-PTH is shown in yellow. LRP2 immunfluorescence is in red. Nuclei are stained with DAPI (blue). Left panels are overlays of all 3 channels. Optical section thickness is 0.5 mm. Scale bar = 20 mm. (E) Proposed mechanism of the LRP2-dependent suppression pathway: LRP2 binds PTH, facilitating exosomal reuptake and lysosomal degradation. Data on the processivity of LRP2 (Megalin)-mediated PTH binding and subsequent lysosomal degradation in mouse T9 cells are based on Hilpert et al (1999) [34].

For LRP2/TMR-PTH confocal experiments, cryopreserved minced tissue from a suppressed parathyroid gland was thawed and divided into 3 aliquots (9, 10, and 10 mg). Each aliquot was incubated in MEM–EBSS–CMF medium supplemented with 1 mM calcium, 0.5 mM magnesium, 0.2% bovine serum albumin, and 20 mM HEPES (pH 7.4). Samples were equilibrated at 4 °C for 30 minutes. 1 mM BSA was added to 2 samples and 1 mM recombinant human RAP protein (Enzo, BML-SE552) was added to the third sample. One BSA-treated aliquot was dosed with 1 µM TMR-labeled human PTH (1-34), while the second BSA-treated aliquot received 1 µM unlabeled PTH (1-34). The RAP-treated sample received 1 mM TMR-PTH. All samples were then incubated for 30 minutes at 37 °C in a 5% CO_2_ incubator. Following incubation, samples were prefixed in 10% formalin for 1 hour at room temperature, washed 3 times with PBS, embedded in OCT compound, and snap-frozen on dry ice. Cryosections (20 µm thick) were prepared and stained immediately with DAPI and Alexa Fluor 488-conjugated wheat germ agglutinin (WGA-488) for 30 minutes to visualize nuclei and membranes, respectively. Sections were washed in PBS and mounted with FluoroShield (Fig. 1C). For the RAP experiments, 20 mM cryosections were prepared and incubated with primary rabbit anti-LRP2 antibodies for 24 hours, followed by incubation with an AlexaFluor 647-conjugated antirabbit secondary antibody for 2 hours. Sections were then counterstained with DAPI. After each incubation step, sections were washed 3 times in PBS and mounted using FluoroShield (Millipore-Sigma, F6182) mounting media.

Confocal microscopy was performed using a Leica DMi8 system and Leica Application Suite software. The TMR detection range was set between 566 and 593 nm to minimize autofluorescence. Z-stacks were acquired at 0.5 µm intervals. Image analysis was carried out using Imaris software with signal cutoff thresholds defined by the control (unlabeled PTH) sample. Z-sections with detectable TMR-PTH signal were exported as TIFF files and independently assessed to confirm subcellular localization relative to plasma membranes (WGA-488 signal) and nuclei (DAPI). TMR-PTH experiment confocal microscoping.

Labeled and unlabeled human PTH (1-34) peptides were generously provided by Dr. Thomas Gardella (Massachusetts General Hospital) and were previously utilized in a published study investigating PTH receptor dynamics [30].

## Results

### snRNAseq profiling of normal human parathyroid glands

Deidentified human parathyroid glands were obtained through our institution's solid organ abdominal transplant service from an unselected sequential series of donors (*n* = 4), using a fully authorized tissue procurement protocol for the recovery of viable, intact parathyroid glands. The parathyroid glands were dissected from en bloc thyroid gland resections using Fluobeam near-infrared wavelength illumination to assist in tissue identification. Parathyroid gland identity was confirmed by histology and by ex vivo PTH production. Glands were recovered from 2 female and 2 male donors, ranging in age from 39 to 56 years old at the time of death (Table 2). Thyroid tissue was collected from 3 of the donors as a comparative reference control.

**Table 2:** Summary of donor characteristics, tissues collected, and nuclei sequenced

Donor ID	Age, sex	Cause of death	Elapsed time from cross-clamp to cell isolation	Cellular viability upon receipt (%)	Tissues collected	Sample ID	Number of nuclei sequenced
1	51, M	GSW	15 hours 09 minutes	93.32 +/− 2.37	Left lower parathyroid	LL(1)	14 630
					Thyroid	T(1)	8360
2	47, F	Cerebral aneurysm	11 hour 45 minutes	89.78 +/− 2.40	Left upper parathyroid	LU(2)	6270
3	39, M	MVA/pedestrian	15 hours 44 minutes	89.66 +/− 4.52	Right lower parathyroid	RL(3)	14 630
					Right upper parathyroid	RU(3)	14 630
					Thyroid	T(3)	6270
4	56, F	Stroke	12 hours 12 minutes	90.78 +/− 6.53	Left lower parathyroid	LL(4-Supp)	12 540
					Left upper parathyroid	LU(4-Supp)	12 540
					Thyroid	T(4-Supp)	10 450

Abbreviations: GSW, gunshot wound; MVA, motor vehicle accident.

**Figure 2:**
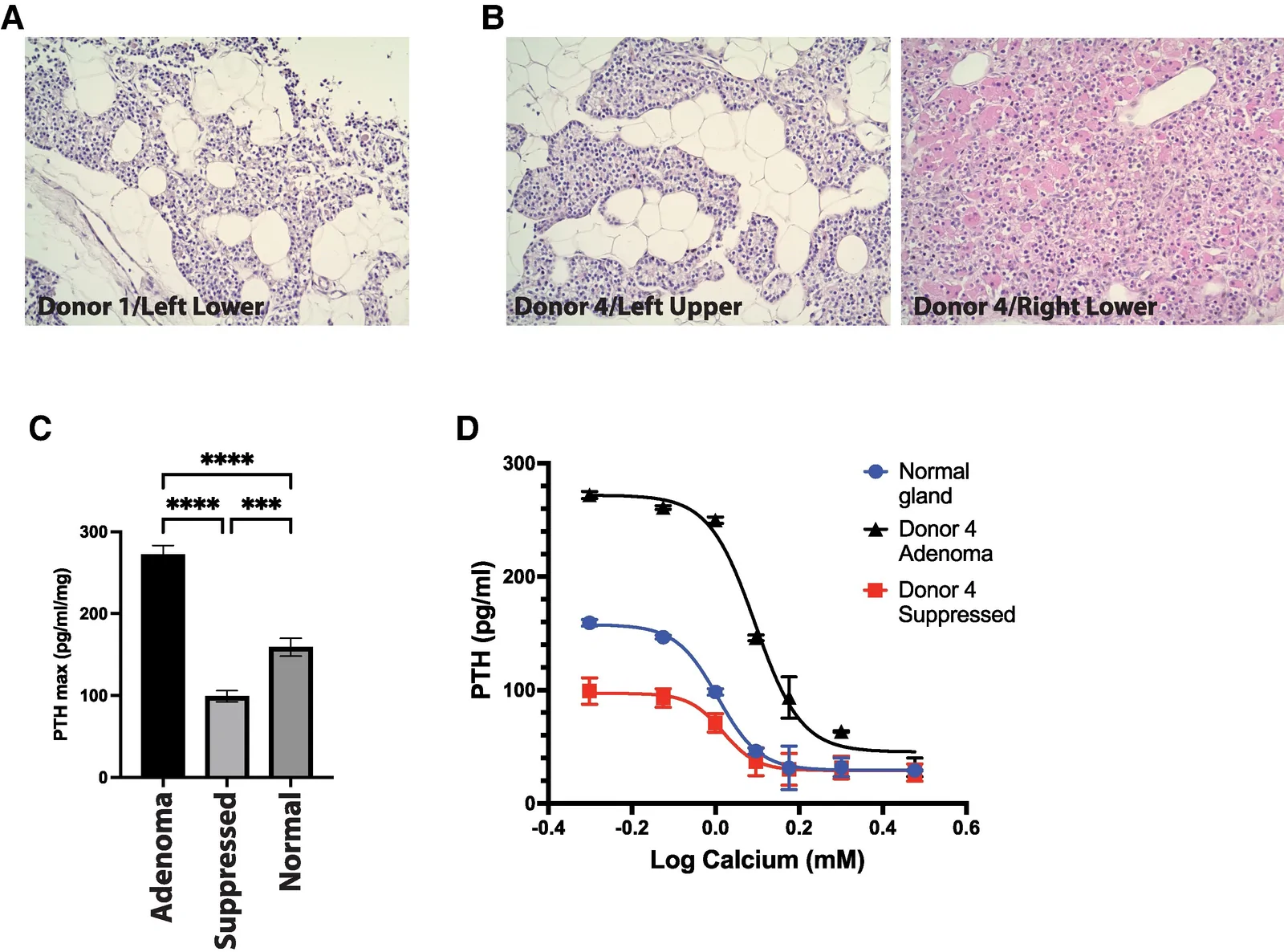
Donor parathyroid gland histological and biochemical characterization. (A) Hematoxylin and eosin (H&E) staining of a normal parathyroid gland from Donor 1 (left lower gland). (B) H&E staining of a suppressed parathyroid gland (left upper) and adenoma (right lower) from Donor 4. (C) Quantification of maximal PTH secretion (pg/mL/15 minutes per 1 mg wet weight of tissue) at 0.5 mM extracellular calcium. Bars represent mean ± SD. Statistical significance was determined by one-way ANOVA with post hoc testing; ***P < .001, ****P < .0001. (D) Normalized PTH secretion setpoint curves from normal glands, Donor 4 adenoma, and Donor 4 normal suppressed glands. Data points are mean ± SD.

Parathyroid glands collected from donors 1, 2, and 3 were histologically normal (Fig. 2A). The right lower gland from donor 4 was grossly enlarged (685 mg; normal range 50-70 mg) and hypercellular (Fig. 2B), consistent with a parathyroid adenoma. The remaining glands from this donor were histologically normal in appearance (Fig. 2B); the left lower and left upper parathyroid glands were processed for snRNAseq. Ex vivo analysis of PTH secretion revealed elevated hormone production by the donor 4 adenoma at 272 pg/mL/15 minutes/mg tissue compared to 159 pg/mL/15 minutes/mg tissue secreted by normal tissue (*P* = .0002). PTH secretion from the suppressed normal gland was 99 pg/mL/15 minutes/mg tissue, substantially reduced relative to the adjacent tumor (*P* < .0001) and significantly depressed relative to reference normal tissue (*P* = .0013), consistent with physiological suppression (Fig. 2C). PTH setpoint analysis revealed an elevated calcium IC_50_ of 1.244 mM (95% CI: 1.198-1.294) in the donor 4 adenoma. In contrast, the donor 4 suppressed gland and the donor 1 normal gland exhibited calcium setpoints within the normal range—1.042 mM (95% CI: 0.9467-1.129) and 1.013 mM (95% CI: 0.9713-1.052), respectively (Fig. 2D). These data demonstrate that the suppressed gland secretes lower tonic levels of PTH but retains normal calcium sensitivity, indicating that the reduced PTH production by the suppressed gland is not simply a consequence of reactively increased CaSR signaling. The suppressed, hyposecretory state of the tumor adjacent to the normal gland is consistent with the common clinical presentation of transient postoperative hypoparathyroidism despite normalized serum calcium levels following resection of a dominant parathyroid adenoma.

Single nuclei suspensions were prepared immediately after procurement using a detergent-based homogenization buffer supplemented with RNase inhibitors to ensure mRNA integrity. Tissue aliquots were collected before and after homogenization for histological examination to confirm the completeness of nuclear extraction and rule out potential selective recovery biases (Fig. S1; Dataset 32209641; https://doi.org/10.6084/m9.figshare.32209641) [16]. Nuclear suspensions were prepared and evaluated by preparative flow cytometry to demonstrate nuclear integrity and isolate singlet nuclei for sequencing (Figs. S2 and S3; Dataset 32209641; https://doi.org/10.6084/m9.figshare.32209641) [16]. Visual assessment of nuclei prior to bar-coding was also performed to confirm input sample quality. cDNA libraries from the isolated nuclei were prepared utilizing the split-pool method [17], following a standard protocol provided by the reagent kit manufacturer.

A total of 83 693 nuclei were sequenced, generating 1.7 billion total paired-end reads. The median number of genes detected per nucleus was 1962, and the mean number of unique molecular identifiers (UMIs) per nucleus was 4609. All samples generated similar mean numbers of UMIs and transcripts per nucleus, indicating consistent and uniform sequencing output (Fig. S4; Dataset 32209641; https://doi.org/10.6084/m9.figshare.32209641) [16]. After sequencing, quality control filtering and preprocessing of sequencing data, the individual sample datasets were integrated and batch-corrected using the Harmony algorithm in Seurat [19].

**Figure 3:**
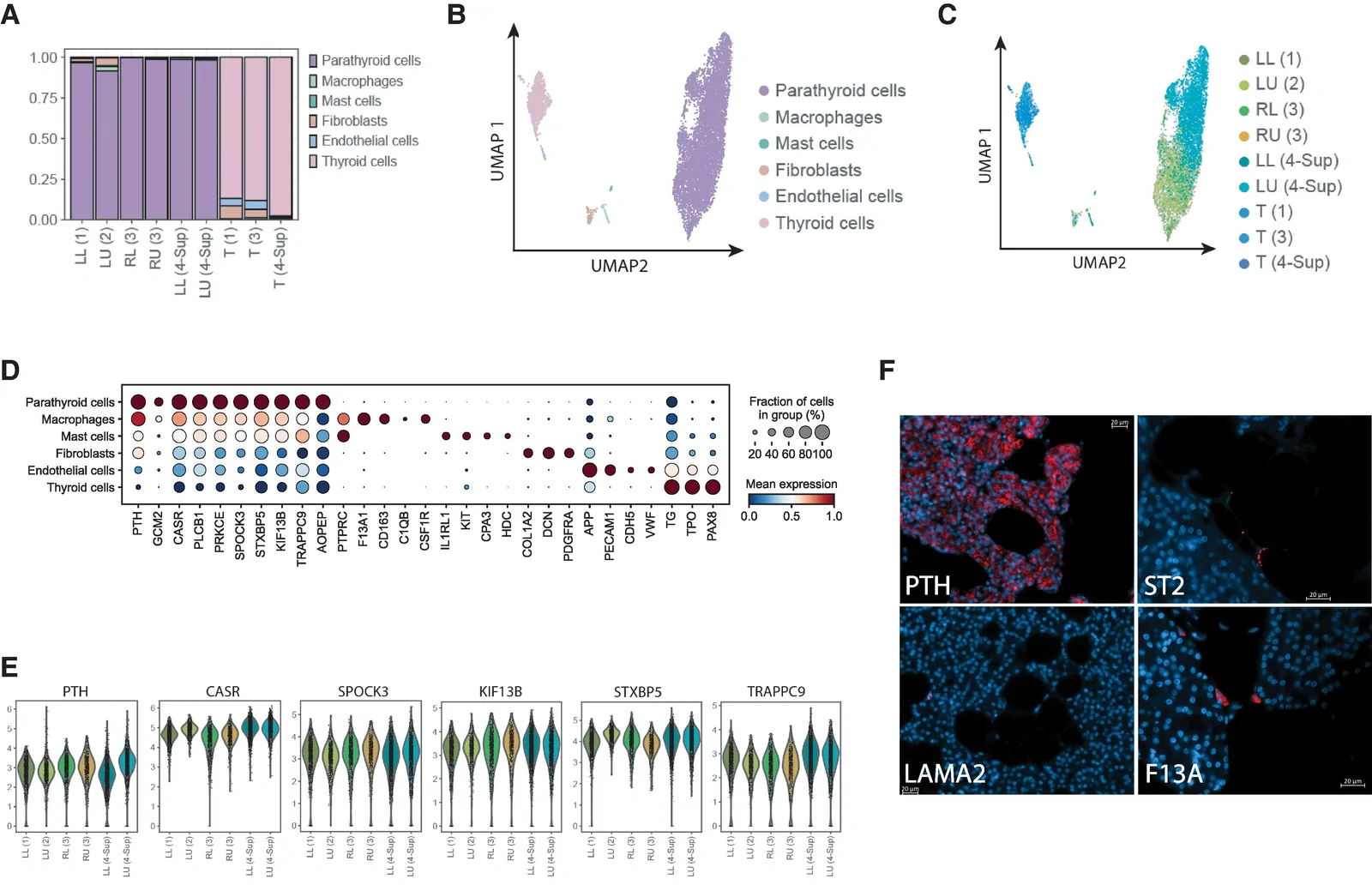
Single-nucleus transcriptomic profiling of human normal and suppressed parathyroid glands. (A) Proportions of major cell populations identified in parathyroid and thyroid samples. (B) UMAP projection of all nuclei, showing the distribution of parathyroid and thyroid cell populations. (C) UMAP projection colored by sample origin (after Harmony integration). (D) Dot plot of selected marker genes across identified cell populations. (E) Violin plots of representative marker genes of parathyroid cells across all parathyroid samples. (F) Representative immunofluorescence images of normal human parathyroid tissue at 40× magnification, staining for PTH, F13A1 (macrophage marker), IL1RL1 (mast cell marker), and LAMA2 (fibroblast marker). Positive immunofluorescent staining is visualized in red. Nuclei stained with DAPI are blue. Scale bar = 20 microns.

Aggregate analysis of the normal and suppressed parathyroid glands and thyroid gland samples revealed 6 major clusters (Fig. 3A and 3B). All 6 clusters were not dependent upon donor origin, as all donor inputs showed substantial overlap (Fig. 3C). The largest group corresponded to parathyroid cells expressing the canonical markers GCM2, PTH, and CASR (Fig. 3D). Thyroid cells, identified by expression of thyroglobulin (TG), thyroid peroxidase (TPO), and paired box 8 (PAX8), segregated as a distinct group. The remaining 4 clusters corresponded to macrophages (CD163/CSF1R positive), mast cells (CD117/HDC positive), fibroblasts (COL1A2/PDGFRA/DCN positive), and endothelial cells (PECAM1/CDH5/VWF positive) based on their respective expression of canonical cell lineage markers (Fig. 3D). The purity of the input specimens demonstrated accurate tissue identification, as more than 85% of nuclei from the parathyroid gland samples corresponded to the parathyroid-specific cluster, while thyroid nuclei accounted for over 90% of the thyroid-specific cluster (Fig. 3A). The parathyroid cluster contained nuclei from both suppressed and normal glands (Fig. 3E). In addition to PTH and the transcription factor GCM2, the calcium sensing pathway components CASR, PLCB1, and PRKCE were equivalently expressed at the transcript level in both suppressed and nonsuppressed normal parathyroid cells, suggesting that physiological suppression was not a consequence of changes in CASR pathway gene expression, consistent with the absence of setpoint changes in suppressed vs nonsuppressed normal glands. In addition, several genes not previously described as parathyroid markers were found to be consistently expressed throughout the entire parathyroid cell cluster. These genes included SPOCK3, STXBP5, KIF13B, AOPEP, and TRAPPC9 (Fig. 3E). In intact parathyroid tissue, the relative abundance of macrophages (visualized by F13A1 immunofluorescence), mast cells (visualized by IL1R1 immunofluorescence), fibroblasts (visualized by LAMA2 immunofluorescence), and endothelial cells (visualized by cytohistology) was consistent with the proportion of cells assigned these identities based on transcriptional signatures (Fig. 3F and Table S1 [16]; Dataset 32209641; https://doi.org/10.6084/m9.figshare.32209641).

**Figure 4:**
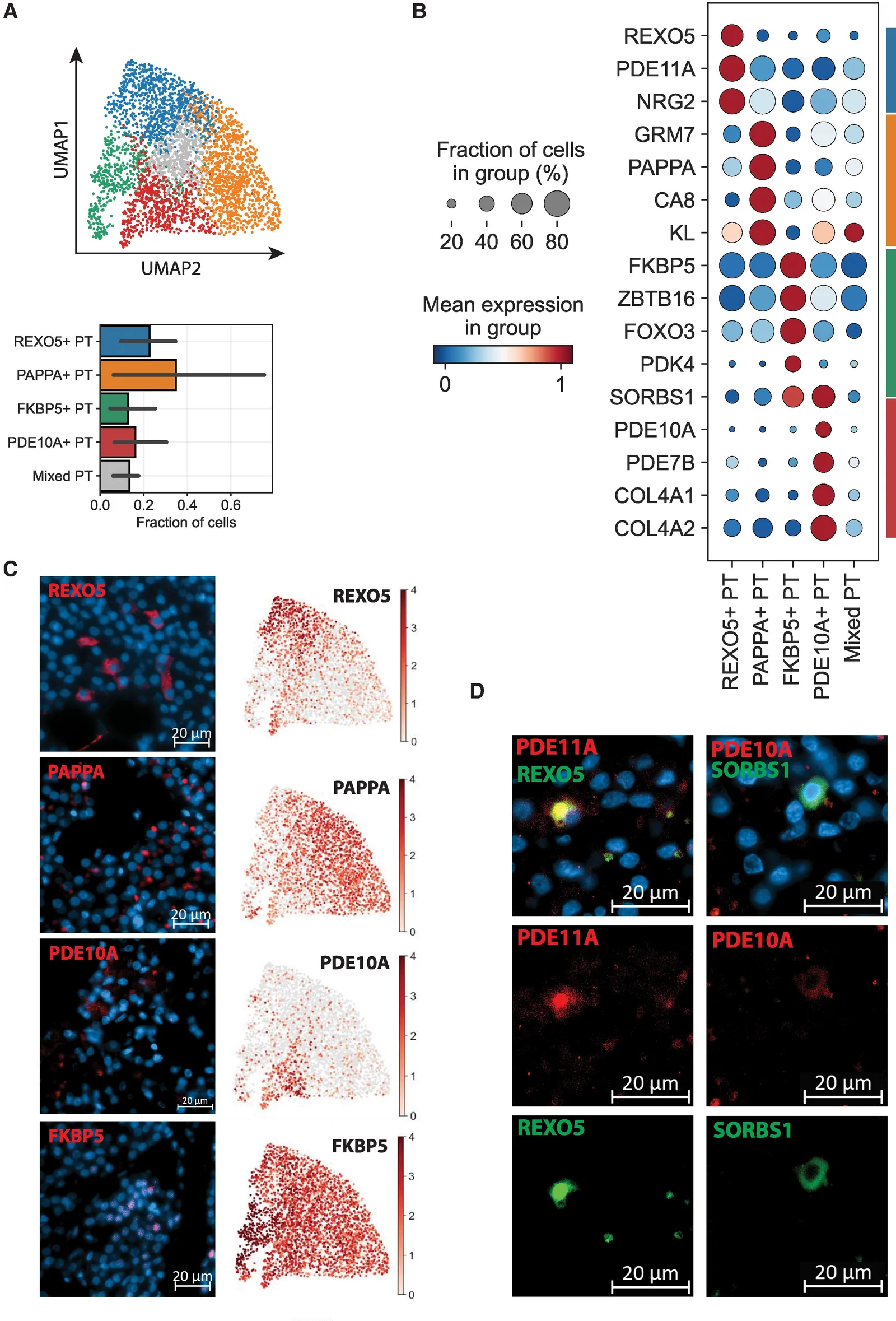
Identification normal human parathyroid gland cell populations. (A) UMAP projection (top) and relative proportions (bottom) of normal human parathyroid cells, revealing 5 transcriptionally defined populations identified by snRNA-seq. Bars represent mean ± SD across parathyroid samples from Donors 1-3. (B) Dot plot showing scaled expression (color intensity) and percentage of expressing cells (dot size) for representative marker genes across the 5 populations. (C) Immunofluorescent staining (left) and UMAP feature plots (right) of key marker genes (REXO5, PAPPA, PDE10A, FKBP5) in normal human parathyroid glands. (D) Colocalization of population-specific markers by immunofluorescence: PDE11A with REXO5, and PDE10A with SORBS1.

To resolve individual signatures within the parathyroid marker group, we analyzed the parathyroid cluster from donors 1-3 independently of the nonparathyroid cell types and the suppressed gland cells. Unsupervised clustering of single nucleus transcriptomes within the parathyroid group revealed 5 transcriptionally distinct populations (Fig. 4A). The first population contained approximately 23% of the parathyroid cells and was defined by high expression of the RNA exonuclease REXO5, the cyclic nucleotide phosphodiesterase PDE11A, and the neuregulin family member NRG2 (Fig. 4B; Dataset 1 [31]). The second population (35% of parathyroid cells) was marked by expression of PAPPA (pregnancy-associated plasma protein A, a metalloproteinase regulating IGF signaling), GRM7 (metabotropic glutamate receptor, a G-protein-coupled receptor), and CA8 (carbonic anhydrase-related protein 8) (Fig. 4B; Dataset 2 [31]). This population also showed the highest expression of KL (Klotho), a protein with known key regulatory functions in PTH secretion and mineral metabolism [32, 33]. This cluster also contained the largest number of differentially expressed markers (65 genes with Log2FC > 1.5, FDR < 10^−5^; Fig. S3). The third population (approximately 13% of the parathyroid cells) was characterized by enhanced expression of FKBP5 (a cochaperone regulating glucocorticoid receptor activity) along with pyruvate dehydrogenase kinase PDK4 and the stress-associated transcription factors ZBTB16 and FOXO3 (Fig. 4B; Dataset 3 [31]). The fourth population specifically expressed the cyclic nucleotide phosphodiesterases PDE10A and PDE7B, as well as basement membrane collagens COL4A1 and COL4A2 (Fig. 4B; Dataset 3 [31]). Finally, we identified SORBS1 as a shared marker of populations 3 and 4. Four of these populations displayed more than 15 robustly enriched marker genes each (genes with Log_2_FC > 1.5, FDR < 10^−5^), while a fifth population designated as mixed parathyroid (Mixed PT) lacked uniquely identifying markers (Fig. 4A and B). With the exception of the REXO5+ population, which was absent in suppressed tissue, we confirmed that the described cellular subtype populations were present in both normal and suppressed glands and could thus be evaluated for comparative analysis of the respective physiological states (Figs. S5–S8 [16]; Dataset 32209641; https://doi.org/10.6084/m9.figshare.32209641)

### Immunofluorescence analysis of marker gene expression

To validate the population-specific markers, we performed immunofluorescent staining on an independent panel of normal parathyroid specimens from seven cadaveric donors (Fig. 4C). Normal parathyroid glands stained positive for REXO5, PAPPA, PDE10A, and FKBP5, with signal restricted to subsets of cells in proportions consistent with transcriptomic data. REXO5 localized to the cytoplasm, appearing either as punctate foci or as diffuse cytoplasmic staining. PDE10A was also detected in the cytoplasm. PAPPA was detected in bright foci associated with the plasma membrane. FKBP5 was detected predominantly in the nucleus with weaker cytoplasmic staining. For the REXO5+ population, we confirmed colocalization of REXO5 with PDE11A (Fig. 4D). We also confirmed colocalization of SORBS1 with PDE10A, marked the PDE10A+ population (Fig. 4D). Based on these observations, we designated the respective parathyroid populations according to their primary marker genes: *REXO5*  *+*  *PT, PAPPA*  *+*  *PT, FKBP5*  *+*  *PT, and PDE10A*  *+*  *PT*.

**Figure 5:**
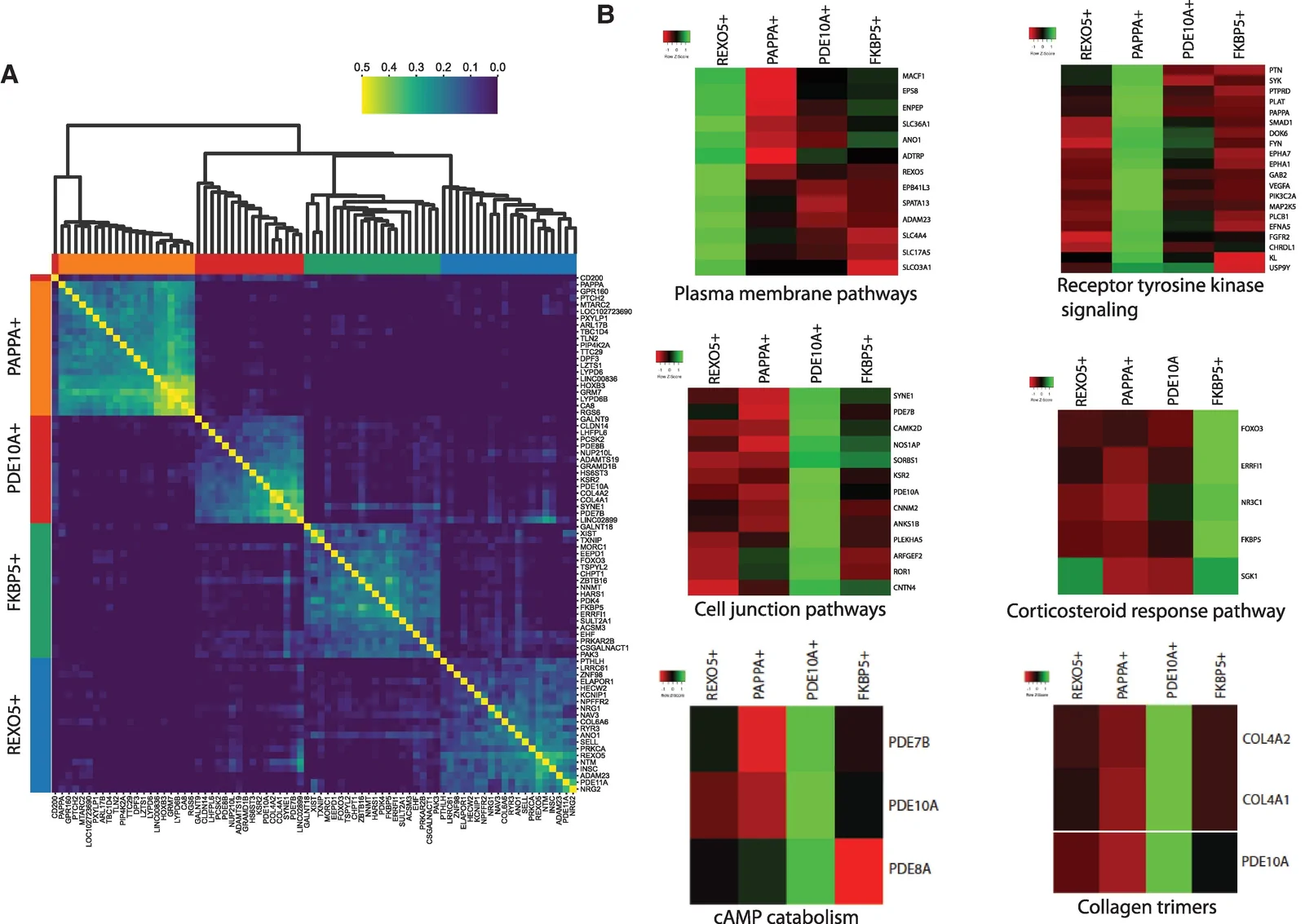
Gene signatures and pathway enrichment in normal parathyroid cell populations. (A) Unsupervised hierarchical clustering and correlation matrix of marker gene expression in normal parathyroid cells across 4 transcriptional clusters (PAPPA+, PDE10A+, FKBP5+, REXO5+). (B) Heatmap of relative marker gene expression across the 4 populations, grouped by 6 enriched Gene Ontology pathways. PDE10A is included in the collagen trimer heatmap as a population-specific marker gene for reference.

To characterize the parathyroid transcriptional clusters functionally, we constructed a correlation matrix to visualize genes that are coordinately regulated within each of the 4 parathyroid populations (Fig. 5A). This analysis revealed strong within-cluster correlations for all 4 of the parathyroid signature clusters. Gene ontology enrichment [25] of the top-scoring genes within each cluster (adjusted *P*-value < 2 e-15, gene score >10) revealed distinct functional pathways uniquely associated with each population (Fig. 5B). For the *REXO5+* signature (39 top genes), we observed enrichment for plasma membrane region pathways (12 of 1333 genes, FDR = 0.0053) (Fig. S9). For the PAPPA+ signature (196 top genes), enriched pathways included aminophospholipid translocation (3 of 7 genes, FDR = 0.037), branching morphogenesis (3 of 6 genes, FDR = 0.03), and cell surface receptor tyrosine kinase signaling (14 of 432 genes, FDR = 0.046) (Fig. S10). The PDE10A+ signature (32 top genes) was enriched for the collagen IV trimer complex (2 of 6 genes, FDR = 0.022) and cell junction pathways (13 of 2417 genes, FDR = 0.021) (Fig. S11). The PDE10A+ cells expressed additional high-activity cAMP phosphodiesterases (PDE7B, PDE8A), consistent with enhanced cAMP catabolic activity in this population. Cells with the *FKBP5+* signature (31 top genes) were enriched for genes in pathways associated with cellular responses to corticosteroid stimulus (4 of 65 genes, FDR = 0.026) (Fig. S12).

### Comparison of normal and suppressed parathyroid gland gene expression signatures

**Figure 6:**
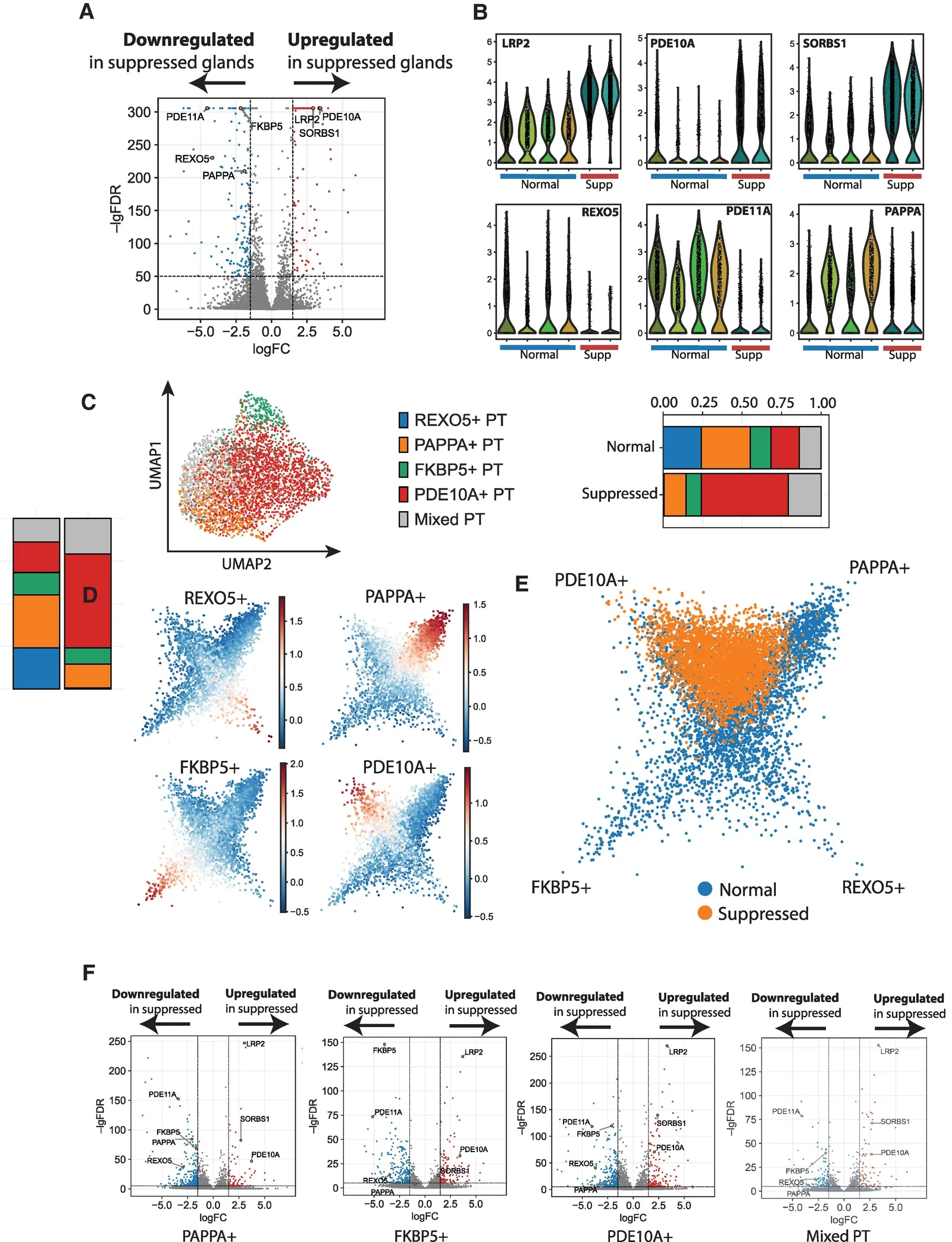
Characterization of the suppressed parathyroid state and its transcriptional divergence from normal tissue. (A) Volcano plot comparing gene expression between suppressed and normal parathyroid cells. Vertical dashed lines indicate |log_2_FC| > 1.5; the horizontal dashed line indicates the significance threshold (−log_10_FDR > 50). Selected up-regulated (PDE10A, LRP2, SORBS1) and down-regulated (PDE11A, REXO5, PAPPA, FKBP5) genes are labeled. (B) Violin plots showing expression of top up-regulated (LRP2, PDE10A, SORBS1) and down-regulated (PDE11A, REXO5, PAPPA) genes in individual parathyroid glands: normal (Donors 1-3) and suppressed (Donor 4). Sample colors correspond to the scheme shown in Fig. 3C. (C) UMAP projection (left) and relative proportions (right) of suppressed human parathyroid cells, revealing redistribution of REXO5^+^, PAPPA^+^, PDE10A^+^, and FKBP5^+^ populations. (D) Gene signatures of the 4 cell populations visualized on tetrahedral UMAP plots. (E) Tetrahedral overlay plot showing redistribution of the 4 population gene signatures (PDE10A^+^, PAPPA^+^, FKBP5^+^, REXO5^+^) in normal (blue) and suppressed (orange) parathyroid cells. (F) Volcano plots comparing gene expression between suppressed and normal samples within the PAPPA^+^, FKBP5^+^, and PDE10A^+^ populations. Vertical dashed lines indicate |log_2_FC| > 1.5; horizontal dashed line indicates the significance threshold (−log_10_FDR > 5). Selected marker genes are labeled.

To investigate the transcriptional characteristics of physiological suppression in normal parathyroid cells, we compared the parathyroid cell expression profiles among the donor 1, 2, and 3 normal glands to the tumor-adjacent suppressed glands from donor 4. Differential expression analysis identified 80 genes that were significantly up-regulated in suppressed glands (log_2_FC > 1.5; FDR = 2 × 10^−50^) and 156 genes that were significantly down-regulated in suppressed glands (log_2_FC < 1.5; FDR = 2 × 10^−50^) (Fig. 6A and B). Suppression was marked by strong induction of the scavenger receptor protein LRP2 (log_2_FC = 3.4), together with up-regulation of SORBS1 (log_2_FC = 2.9) and PDE10A (log_2_FC = 3.4), the latter a defining marker of the PDE10A+ population. In contrast, markers of the other populations (REXO5 (log_2_FC = −4.2), PDE11A (log_2_FC = −4.5), PAPPA (log_2_FC = −1.9), and FKBP5 (log_2_FC = −2.2)) were prominently down-regulated in suppressed tissues (Fig. 6A and 6B). To assess the effect of suppression on cellular composition, we projected the distribution of the normal parathyroid cell populations onto a UMAP plot of the suppressed gland transcriptional profile (Fig. 6C). Suppression resulted in a near-complete loss of REXO5^+^PT cells, reduction of FKBP5^+^PT and PAPPA^+^PT cells, a modest increase in the Mixed PT population, and a striking expansion of the PDE10A^+^PT population to >50% of the overall parathyroid cell population (Fig. 6C). To visualize this transcriptional profile redistribution, we constructed a tetrahedral plot of normal gland nuclei with apical vertices representing the 4 major populations (Fig. 6D). Suppressed cells shifted strongly toward the PDE10A vertex, accompanied by marked loss of REXO5+ and FKBP5+ signatures. Notably, cells with a PAPPA+ signature partially shifted toward the PDE10A+ signature, suggesting coexpression or a potential transition between the 2 states (Fig. 6E). Independent differential expression analysis within the PDE10A^+^PT, PAPPA^+^PT, and FKBP5^+^PT populations showed consistent induction of LRP2 and PDE10A, indicating a shared transcriptional program engaged during physiological suppression regardless of population of origin (Fig. 6F). Conversely, down-regulated genes showed greater population specificity; PAPPA expression diminished predominantly in PAPPA+ cells, and FKBP5 was down-regulated in its respective cluster. REXO5 and PDE11A expression declined across all clusters. Given the near-complete loss of the REXO5^+^ population in suppressed glands, direct comparison with its normal counterpart was not feasible. These transcriptional shifts were further reflected in pathway-level analysis. The top up-regulated genes in suppressed glands included components of stromal network-forming collagen trimers (3 genes, FDR = 0.0037) and signal transduction processes (47 genes, FDR = 0.041), the latter category encompassing the most significantly up-regulated genes (LRP2, PDE10A, and SORBS1) (Fig. S13). Among the top down-regulated genes in suppressed glands, we observed significant enrichment for pathways related to cell junction organization (60 genes, FDR = 0.00001), plasma membrane organization (25 genes, FDR = 0.003), adherens junction components (10 genes, FDR = 0.019), and small molecule binding (105 genes, FDR = 0.048) (Dataset 5 [31], Fig. S14)). These data suggested that suppression preferentially attenuates normal transcriptional programs, particularly those associated with the REXO5+ population, while inducing a distinct, shared program characterized by LRP2 and PDE10A activation.

### Immunofluorescence comparison of suppressed and normal parathyroid glands

**Figure 7:**
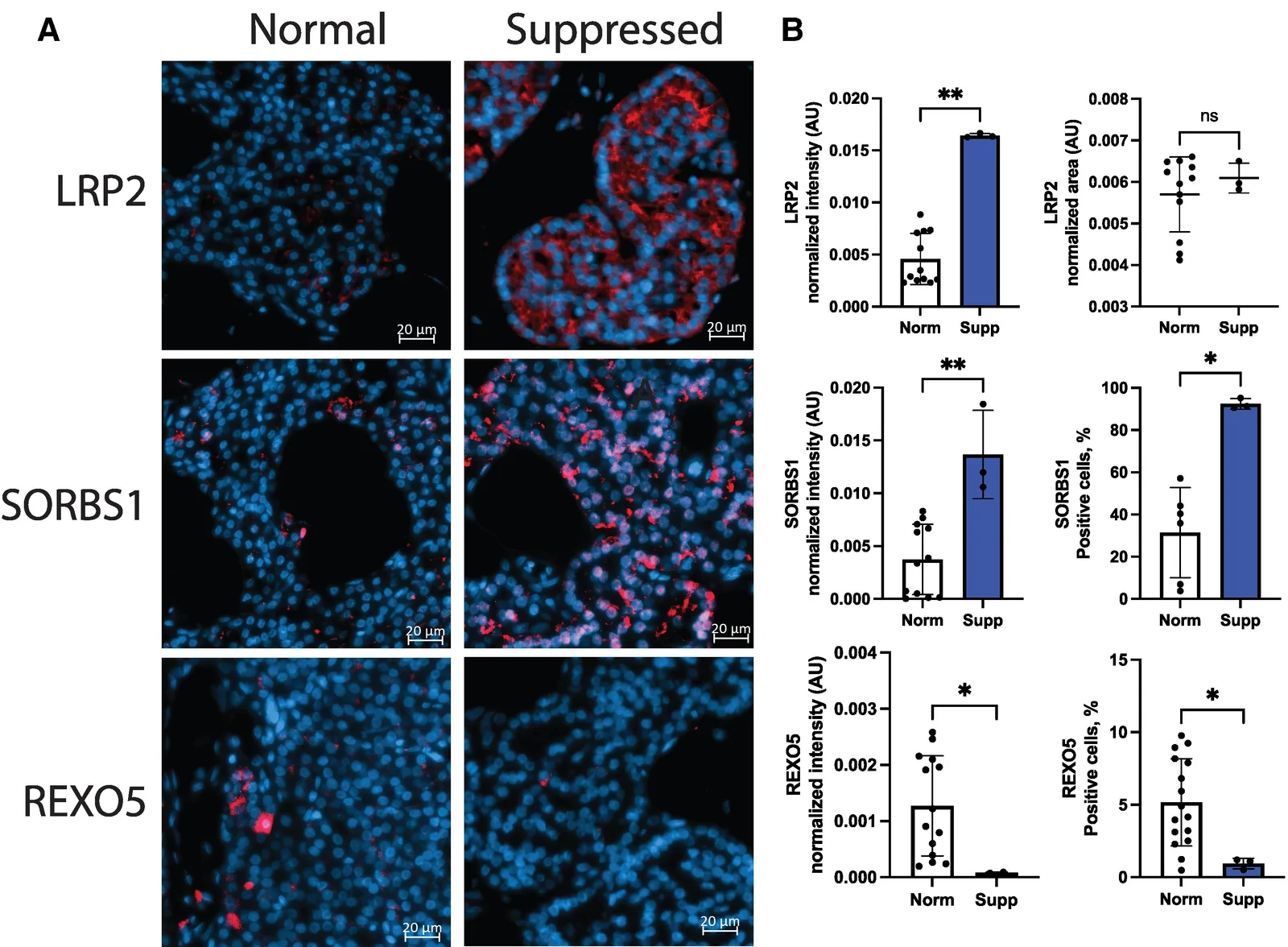
Immunofluorescent characterization of protein expression in normal and suppressed donor 4 parathyroid glands. (A) Immunofluorescent staining of LRP2, SORBS1, and REXO5 in independent normal and suppressed (Donor 4) parathyroid tissue. Nuclei are counterstained with DAPI. (B) Quantification of protein expression by immunofluorescence: mean fluorescence intensity normalized to nuclear count (arbitrary units, AU) for LRP2, SORBS1, and REXO5 (left), normalized fluorescence area for LRP2 (right top), and percentage of positive cells for SORBS1 and REXO5 (right bottom). Data represent mean ± SD. Statistical significance was determined using the Mann–Whitney test (*P < .05; **P < .01).

We next performed immunofluorescent staining for LRP2, SORBS1, REXO5 (Fig. 7), and PAPPA (Fig. S15 [16]; Dataset 32209641; https://doi.org/10.6084/m9.figshare.32209641) in an independent cohort of normal parathyroid glands in comparison to the suppressed glands from donor 4. LRP2 (also known as Megalin) was detected in nearly all parathyroid cells in both normal and suppressed glands, with no difference in normalized positive area, but with a 7-fold increase in normalized intensity in the suppressed glands (*P* = .0044; Fig. 7A and 7B). LRP2 staining localized to membranes and the cytoplasm of the majority of parathyroid cells. SORBS1 also showed significant induction in suppressed glands, with a 3.5-fold increase in normalized intensity (*P* = .0044) and an expansion in the proportion of positive cells from 31% in normal glands to 92% in suppressed glands (*P* = .024). In contrast, REXO5 showed a 15-fold reduction in staining intensity (*P* = .0017), with the proportion of positive cells decreasing from 5.7% to 0.9% (*P* < .014), consistent with the transcriptomic findings. PAPPA staining intensity and frequency were unchanged (40% in normal vs 38% in suppressed glands; Fig. S15 [16]; Dataset 32209641; https://doi.org/10.6084/m9.figshare.32209641).

To validate these observations, we assembled an independent cohort of suppressed gland samples from patients who underwent parathyroidectomy with curative intent for PHPT. We conducted a retrospective search for PHPT parathyroidectomy cases at our institution in which 2 or more glands were removed, with at least one gland subsequently found to be normocellular and uninvolved with the culprit adenoma. Six cases meeting these criteria were identified (Table 1). Review of histopathological images and intraoperative PTH (ioPTH) data confirmed the designation of the normocellular and adenoma status of the respective resected glands. These data are summarized in Table 1 and Table S2 [16] (Dataset 32209641; https://doi.org/10.6084/m9.figshare.32209641).

**Figure 8:**
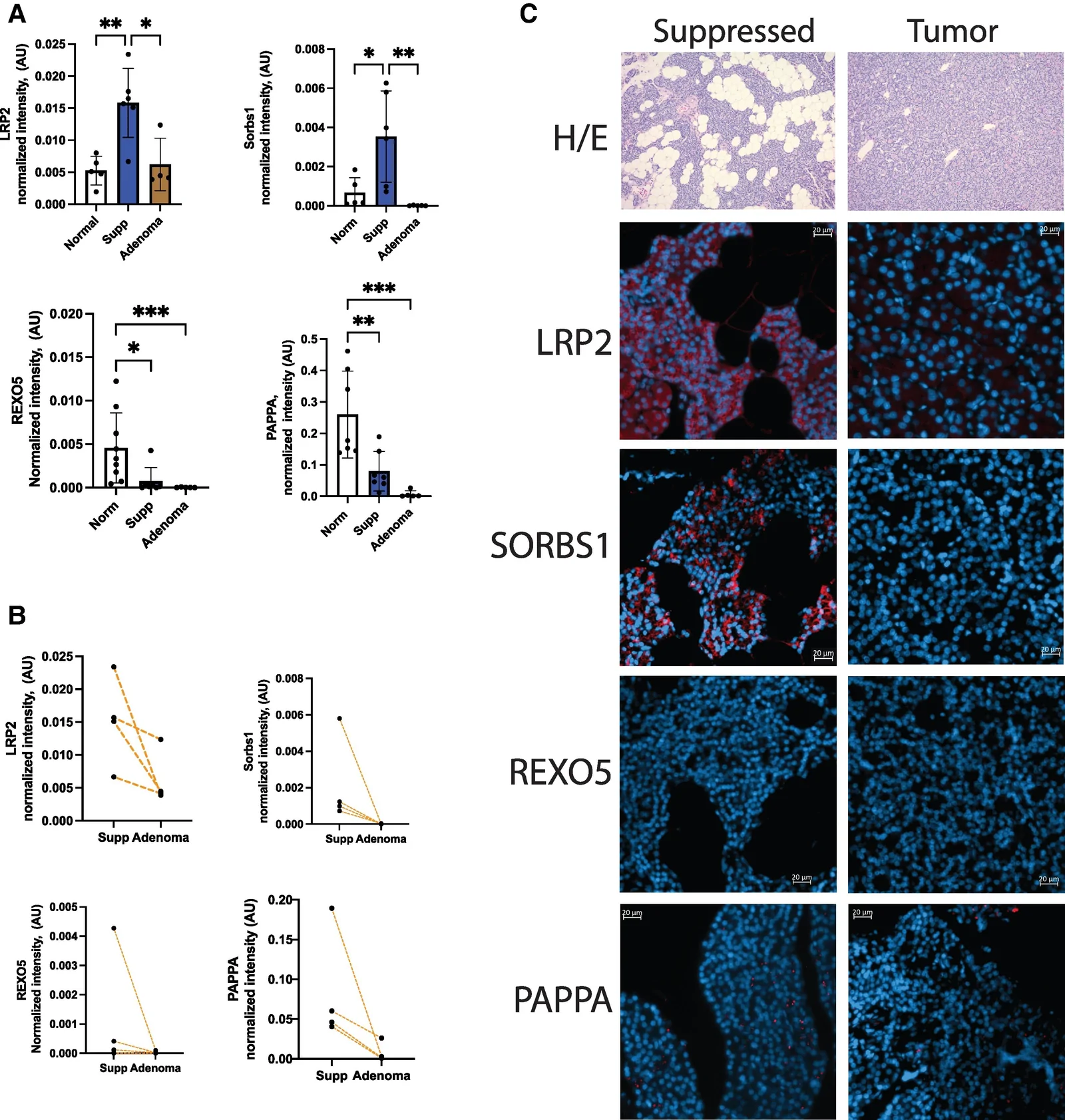
Immunofluorescent characterization of protein expression in normal tissue, suppressed parathyroid glands, and adjacent adenomas. (A) Quantification of protein expression by immunofluorescence: mean fluorescence intensity normalized to nuclear count (arbitrary units, AU) for LRP2, SORBS1, REXO5 and PAPPA. Data represent average fluorescence intensity, mean ± SD. Statistical significance was determined using one-way ANOVA with post hoc testing (*P < .05; **P < .01; ***P < .01). (B) Paired comparison of LRP2, SORBS1, REXO5 and PAPPA protein expression in suppressed glands vs their adjacent adenomas. (C) Hematoxylin and eosin staining and immunofluorescent labeling for LRP2, SORBS1, REXO5, and PAPPA in suppressed glands and their adjacent adenomas. Nuclei are counterstained with DAPI in the immunofluorescent images.

We compared the suppressed glands from this group to their case-matched adenomas and to reference normal tissue (Fig. 8). For LRP2, suppressed glands showed a 2-fold increase in staining intensity relative to both normal glands (*P* < .01) and their paired adenomas (*P* < .05), consistent with the transcriptomic data. LRP2 abundance in adenoma cells was similar to normal glands. SORBS1 staining demonstrated a similar expression pattern. SORBS1 expression was increased in suppressed glands relative to normal glands and was nearly absent in adenomas. Paired comparisons of case-matched suppressed glands and adenomas demonstrated consistent down-regulation of suppression-associated pathways in adenomas from all cases in the confirmatory cohort. REXO5 staining was nearly undetectable in both suppressed glands (*P* < .05) and adenomas (*P* < .01) relative to normal parathyroid glands. PAPPA staining intensity was decreased in both suppressed glands (3-fold, *P* < .01) and adenomas (40-fold, *P* < .001) compared to normal tissue, with adenomas demonstrating reduced signal in all case-matched comparisons.

### Inhibition of suppression-induced factors relieves down-regulation of PTH secretion

To test the concept that increased LRP2 and PDE activity could contribute to reduced PTH production in suppressed parathyroid glands, we assessed the effects of inhibiting these proteins on ex vivo PTH secretion in 4 specimens of live human parathyroid tissue [29]. We evaluated PTH secretory responses in fresh donor normal parathyroid glands (clear bars, Fig. 1A), and cryopreserved, histologically normal suppressed glands (blue bars, Fig. 1B). To inhibit LRP2, we utilized an antibody that binds an epitope between amino acids 4446-4655, sterically obstructing a domain previously shown to be essential for maintaining the receptor in a ligand-receptive conformation [34-36]. We preincubated intact parathyroid tissue for 30 minutes with 120 ug/mL of the inactivating LRP2 polyclonal antibody (PA5-92032, ThermoFisher, RRID:AB_2806358) or an equivalent concentration of purified rabbit IgG. In parallel, PTH production was measured without antibody addition (VEH). PTH secretion under each condition was determined by quantitating the amount of PTH produced over 15 minutes in the presence of 0.5 mM calcium to stimulate maximal PTH release. PTH concentrations were normalized to tissue mass, and each experiment was conducted in triplicate. Exposure to the inactivating LRP2 antibody induced a significant increase in maximal PTH secretion (253.6 pg/mL/15 minutes/mg) over the no antibody (168.9 pg/mL/15 minutes/mg) and control IgG (116.9 pg/mL/15 minutes/mg) conditions (*P* = .0012 for difference by ANOVA), consistent with relief of LRP2 antagonism with respect to PTH secretion (Fig. 1A) Cryopreserved suppressed tissue treated with the LRP2-blocking antibody exhibited a similar response, showing a significant 10% increase in PTH secretion relative to control (Fig. 1B). Because suppressed glands were analyzed following cryopreservation while normal donor glands were studied without prior cryopreservation, absolute PTH secretion values between these groups are not directly comparable. Therefore, functional effects were interpreted using relative changes normalized to matched control tissue within each experimental condition.

LRP2 is a well-characterized scavenger receptor that mediates uptake of various metabolites followed by endocytosis, and PTH is among its known ligands [34]. To investigate whether parathyroid tissue can reabsorb extracellular PTH, we incubated cryopreserved suppressed parathyroid tissue with a bioactive form of synthetic PTH conjugated with the red fluorescent moiety tetramethylrhodamine (TMR-labeled PTH(1-34)) [30]. Using confocal microscopy, we detected both membrane-localized TMR-PTH foci and intracellular cytoplasmic accumulation of labeled PTH (Fig. 1C). Pretreatment of parathyroid tissue with the receptor-associated protein RAP, a highly specific inhibitor of LRP2 ligand binding [37], markedly reduced TMR-PTH uptake compared to coincubation with BSA as a negative control (Fig. 1D, Fig. S16). These observations of extracellular PTH internalization by parathyroid tissue are consistent with an active scavenger function of LRP2 during physiological suppression (Fig. 1E).

**Figure 9:**
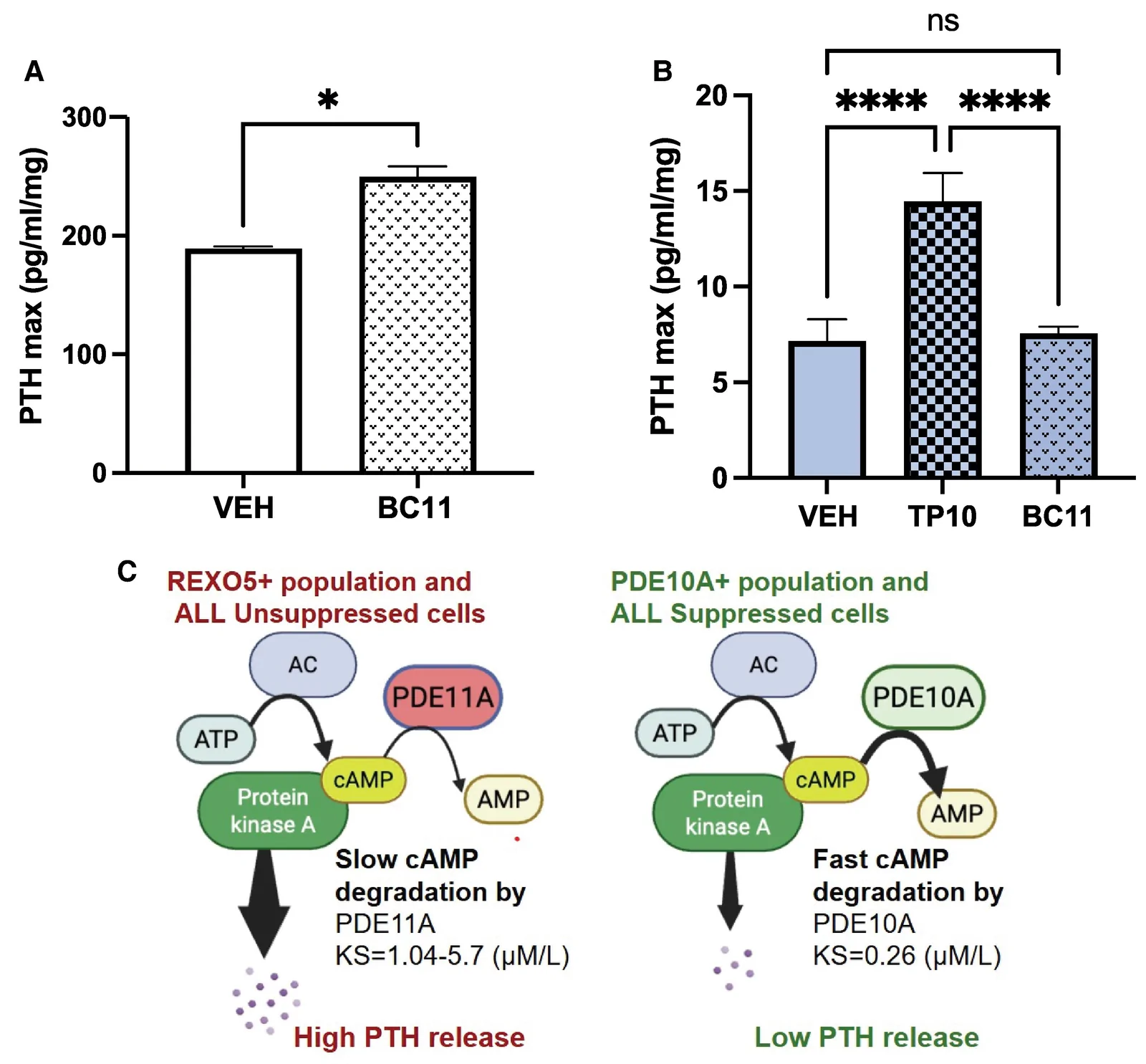
Inhibition of PDE activity enhances maximal PTH secretion in a subtype-specific manner. (A) Normal tissue in the presence of the highly selective PDE11 inhibitor BC11-38 (10 µM) or DMSO (VEH). (B) Suppressed tissue in the presence of the highly selective PDE11 inhibitor BC11-38 (10 µM) or TP10 (100 nM). (C) Schematic model of cAMP degradation and PTH release in REXO5^+^ unsuppressed cells (left) and PDE10A^+^ suppressed cells (right). Slow cAMP degradation by PDE11A in unsuppressed cells is associated with high PTH release, whereas rapid cAMP degradation by PDE10A in suppressed cells results in low PTH release. Data on phosphodiesterase processivity are from Omori and Cotera (2006) [39], and the relationships of adenylyl cyclase and protein kinase A with PTH secretion are from Conigrave (2016) [38].

Because cAMP is a well-recognized key driver of PTH secretion [38], we hypothesized that a switch from PDE11A, a slower acting (*K*_s_ = 1.04-5.7 mM/L) phosphodiesterase [39, 40] predominant in unsuppressed cells, to PDE10A, a faster acting (*K*_s_ = 0.26 mM/L) phosphodiesterase [39, 40] predominant in suppressed cells, would have the effect of reducing PTH release by increasing turnover of cAMP to AMP. To test this idea, we treated normal parathyroid tissue with BC11-38 (BC11), a highly selective, potent, and biologically active inhibitor of PDE11 [41, 42]. BCE11 induced a significant increase in PTH production in our ex vivo system [29], from 188.3 pg/mL/15 minutes/mg in the vehicle negative control to 249.3 pg/mL/15 minutes/mg in the presence of the inhibitor (*P* = .0117) (Fig. 9A). BC11 treatment had no effect on PTH secretion in suppressed glands (Fig. 9B), consistent with reduced PDE11A expression and activity in suppressed cells. In contrast, the selective PDE10 inhibitor TP10 [43] induced a 2-fold increase in PTH secretion in suppressed tissue (Fig. 9B), consistent with a role for PDE10A in mediating reduced PTH secretion in the suppressed state.

Consolidating these results with the expression data, we propose 2 complementary modes of action through which suppressed normal parathyroid glands can reduce tonic PTH hypersecretion independent of CaSR-mediated setpoint signaling. In one mechanism, the increase in LRP2 abundance in suppressed cells could enhance autocrine binding of PTH by this scavenger receptor, with subsequent exosomal reabsorption and lysosomal degradation of the peptide hormone (Fig. 1D). This model is supported by evidence of internalization of extracellular TMR-PTH, the inhibition of TMR-PTH uptake upon LRP2 inhibition, and the reversal of PTH secretory levels following LRP2 antibody blockade (Fig. 1A-1D). In parallel, the switch from PDE11A-expressing normal cells to the faster acting PDE10A phosphodiesterase in suppressed cells could accelerate cAMP turnover and thereby reduce PTH release (Fig. 9C). Although further studies are warranted to fully define these novel mechanisms, the conceptual framework of identifying potentially targetable pathways natively employed in normal suppressed parathyroid tissue to mitigate PTH hypersecretion could lead to alternative, nonsurgical approaches for managing hyperparathyroidism and calcium homeostasis.

## Discussion

In this study, we performed a single-nuclei RNAseq analysis of normal human parathyroid glands to identify the constituent cell types that comprise healthy parathyroid tissue. During procurement, we discovered that one of the presumptive normal donors in our sample set had developed single gland PHPT, leaving the 3 remaining normal glands functionally suppressed. This unexpected finding provided us the opportunity to evaluate functionally suppressed glands and identify transcriptional programs natively employed by normal parathyroid tissue to reduce PTH secretion in the presence of an adjacent, dominant adenoma. By comparing true normal glands to normal suppressed glands, we were able to show that suppression coincides with a shift in the relative abundance of the 4 parathyroid populations observed in unperturbed normal tissue, resulting in a switch between predominant phosphodiesterase subtypes and activation of the receptor LRP2, a scavenger protein known to bind PTH and competitively interfere with PTHR1 downstream signaling [34].

Our approach of characterizing normal donor parathyroid glands addresses an important knowledge gap in the field, as transcriptome studies of human parathyroid tissue to date have focused primarily on pathological conditions such as adenomas [44, 45], secondary hyperplasias [46, 47], parathyroid carcinomas [48], and atypical parathyroid tumors [49]. Due to the challenges of obtaining viable, normal human parathyroid tissue for analysis, molecular studies of healthy parathyroid glands have been limited primarily to animal models [50]. As a consequence, our current understanding of heterogeneity in the cellular composition [4, 5] and functional behavior [9-11] of parathyroid cell types among tumors has not extended to molecular characterization of normal human tissue to date. Our dataset from normal glands reveals uniform expression of a core group of genes not previously associated with the parathyroid but which have functional links to calcium homeostasis and secretory processes in other cell types. SPOCK3, an extracellular calcium-binding proteoglycan highly expressed in the brain, contributes to extracellular matrix-associated calcium buffering [51]. Several additional genes enriched in the normal parathyroid are implicated in vesicular transport and regulated secretion. STXBP5 encodes a syntaxin-binding protein that modulates Ca++-dependent exocytosis in platelets [52]; KIF13B is a plus-end-directed kinesin motor mediating microtubule-based vesicle transport in photoreceptors [53]; TRAPPC9 functions as part of the TRAPP II complex required for endosome-to-Golgi vesicular trafficking in the kidney, heart, and brain [54]; and AOPEP encodes a zinc-dependent aminopeptidase involved in proteolytic activation of peptide hormones in the vascular endothelium [55]. The expression of these genes suggests that the parathyroid gland may incorporate multiple mechanisms linking calcium levels to cellular secretory activity, utilizing components not previously understood to be active in this setting.

The snRNAseq data presented here show that 4 transcriptome profiles can be distinguished within the overall cohort of PTH-expressing cells isolated from normal human parathyroid glands. The PAPPA+ cluster, the most abundant of the 4 subpopulations, is enriched for genes involved in morphogenesis and membrane dynamics, including receptor tyrosine kinase signaling. PAPPA+ cells are notable for their expression of Klotho (KL), a coreceptor enabling FGF23 to signal through FGFRs in the parathyroid, where this pathway is known to regulate PTH secretion [56, 57]. PAPPA itself encodes the secreted protease PAPP-A, which can potentiate IGF-1 signaling by releasing IGF-1 from inactivating carrier proteins [58]. Other genes enriched in the PAPPA+ signature include *GRM7* (metabotropic glutamate receptor 7), a GPCR that activates MAPK/cAMP pathways downstream of glutamate [59], and *CA8* (carbonic anhydrase 8), a catalytic subunit that inhibits IP3-receptor–mediated Ca^2+^ release [60].

Functional gene ontology analysis of the REXO5+ population revealed significant enrichment of membrane-associated components, including the transporters *SLCO3A1* and *SLC36A1*, the ion channel *ANO1*, the adaptor protein *EPS8,* and the peptidases *ADAM23* and *ENPEP*. Enhanced expression of these genes suggested that REXO5+ cells may play a central role in signal integration and vesicular trafficking at the cell periphery. REXO5 encodes a 3′ to 5′ RNA exonuclease with a well-characterized role in the maturation of male germ cells, where it localized to the nucleus and was required for spermatogenic differentiation [61]. Published transcriptomic datasets confirmed that REXO5 expression is almost entirely restricted to the testis, with only low-to-moderate expression in the parathyroid gland [62]. The unexpected presence of REXO5 in parathyroid glands from both male and female donors suggested a previously undefined function for this enzyme; notably, while REXO5 is restricted to the nucleus in the testis, in the parathyroid gland, staining was found in the cytoplasm, possibly indicating tissue-specific compartmentalization of REXO5 activity. The REXO5+ population was characterized by high expression of PDE11A and low levels of high-processivity phosphodiesterases expressed in PDE10A+ cells. Physiological suppression corresponded to a near complete loss of the REXO5+ cluster as shown by both snRNAseq and immunohistochemical analysis.

The PDE10A+ population expressed multiple high-affinity cAMP phosphodiesterases (*PDE10A, PDE7B, and PDE8A)* [63] and showed enrichment for extracellular matrix components, including type IV collagen genes (*COL4A, COL4A2*) [64]. PDE10A+ cells also expressed the adaptor protein *SORBS1*, implicated in insulin- and glucose-related signaling in other secretory tissues [65, 66]. These features suggested that PDE10A+ cells maintain a transcriptomic program that combines rapid cAMP degradation with extracellular matrix remodeling, consistent with a secretory cell state characterized by low cAMP activity.

In normal glands suppressed by an adenoma, the relative abundance of the transcriptional subpopulations changed significantly, with the PDE10A signature group becoming the predominant cohort while the other 3 populations were substantially diminished (Datasets 6-9 [31]). This shift coincided with robust up-regulation of PDE10A and SORBS1 (log_2_FC = 3.4 and 2.9; *P* < 10^−300^), implicating these genes in suppression-associated transcriptional changes. The selective induction of PDE10A and SORBS1 suggested that attenuated PTH release could be mediated by accelerated cAMP degradation. Tonic PTH secretion was strongly down-regulated in the suppressed glands while the calcium/PTH setpoint remained unchanged, suggesting a modulation in sustained secretory activity rather than an alteration in calcium-sensitive signaling as the driver of reduced PTH production in this context. Consistent with this idea, the expression levels of CASR, PLCB1, and PRKCE—key components of CaSR-mediated signaling—were maintained in both suppressed and unsuppressed glands. Immunofluorescence imaging of markers associated with each parathyroid population in an independent set of archival normal parathyroid glands confirmed the presence of cells with the corresponding transcriptome profiles in proportions consistent with the snRNAseq data. Colocalization studies revealed that PDE11A was confined to REXO5^+^ cells, distinct from the PDE10A^+^ subset. Suppressed glands from Donor 4, as well as the independent cohort of suppressed glands, displayed increased LRP2 and SORBS1 staining intensity, a higher proportion of SORBS1^+^ cells, and a pronounced loss of REXO5^+^ cells, consistent with the transcriptomic data and reinforcing the link between suppression and subpopulation remodeling. In contrast, primary adenomas showed an almost complete loss of staining intensity for the population markers SORBS1, PAPPA, and REXO5 compared to suppressed glands (Fig. 8), suggesting a collapse of the normal parathyroid cell subpopulation structure and a potential loss of specialized transcriptional identities in adenomatous tissue.

Comparative Gene Ontology analysis of the pathways associated with the 4 parathyroid populations in normal and suppressed glands suggested that cytoskeletal structural changes may be associated with the transition between these 2 activity states. Components associated with the cellular periphery, including adherens junctions, cell junction organization, and aminophospholipids, were down-regulated in suppressed glands. Conversely, components associated with collagen heterotrimers, which form three-dimensional nets with basement membranes, were up-regulated in suppressed glands. The most highly up-regulated genes in suppressed glands were LRP2, PDE10A, and SORBS1, which are linked to signal transduction processes at the cell membrane (LRP2) [36], cAMP second messenger regulation (PDE10A) [67], and adaptor protein function in signaling and focal adhesion structures (SORBS1) [68]. Enrichment of these respective pathways in the differentially expressed genes suggested that cytoskeletal remodeling may accompany the reduction in PTH secretion in suppressed parathyroid glands, along with enhancement of scavenging receptor activity and modulation of cAMP turnover.

Intracellular cAMP is a central regulator of PTH secretion: early studies showed that G_s_-coupled agonists elevate cAMP and stimulate PTH release, while high extracellular calcium suppresses cAMP via CaSR activation [21, 69, 70]. More recently, an optogenetic approach in human parathyroid tissue demonstrated that reducing cAMP via light-driven Ca++ influx and membrane depolarization led to immediate, reversible inhibition of PTH secretion [71]. Despite the well-established role of cAMP signaling in parathyroid physiology, relatively little is known about the endogenous phosphodiesterases that regulate cAMP levels in the parathyroid gland. Our data suggest that PDE10A is transcriptionally induced in suppressed glands adjacent to PHPT-driving hypersecretory parathyroid adenomas, possibly as an adaptive response to chronic hypercalcemia. In this context, sustained CaSR signaling may lower cAMP both acutely (via Gi pathways) and chronically (via PDE induction), providing dual control over secretion. A switch from PDE11A^+^ cells to cells expressing the faster-acting PDE10A in suppressed tissue would enhance cAMP hydrolysis, reducing PTH secretion. Our data are consistent with these respective roles for PDE11A in normal tissue and PDE10A in suppressed glands, supporting dynamic switching of PDE expression as a novel response mode for regulating PTH secretion.

The marked increase in LRP2 abundance in suppressed glands suggests a second potential mechanism for mitigating PTH hypersecretion in normal, tumor adjacent parathyroid glands. Enhanced expression of LRP2 in suppressed glands could activate an autocrine scavenging mechanism, where parathyroid-expressed LRP2 can bind and internalize locally produced PTH, capturing the hormone locally and reducing overall PTH levels. LRP2 is expressed at low but detectable levels in normal glands, suggesting that this pathway may likely operate under normal physiological conditions as well. Our LRP2 inhibition data and evidence of active uptake of extracellular PTH by parathyroid tissue are consistent with this model, although confirmation will await additional mechanistic experiments to prove the role of LRP2 in direct binding and endocytic degradation of secreted PTH.

The notion that PTH hypersecretion could potentially be mitigated by activating endogenous pathways natively employed by normal parathyroid tissue raises the prospect of nonsurgical interventions to treat hyperparathyroidism patients as a potential alternative to parathyroidectomy. Patients with secondary hyperparathyroidism (SHPT) caused by chronic kidney disease (CKD) are often managed medically with calcimimetics rather than surgery due their significant comorbidities, multi-gland involvement, and overall complexity of disease. Calcimimetics remain an imperfect solution, as these agents induce moderate PTH suppression and carry potential risks of causing hypocalcemia, hyperphosphatemia, gastrointestinal issues, and increased calciuria [72, 73]. Since CKD-associated SHPT hyperplasia is believed to be driven by the physiological milieu of renal failure rather than cellular transformation, it is plausible that native regulatory mechanisms in these parathyroid glands could remain largely intact. If the biochemical pathways driving normal gland suppression can be elucidated in greater detail, it may be possible to activate endogenous mechanisms for the mitigation of PTH hypersecretion for the medical management of SHPT patients.

Limitations of this study include the small primary donor cohort, the indirect nature of the inhibitor assays assessing the consequences of LRP2 and PDE11A inhibition, and the absence of direct comparisons between suppressed glands and parathyroid adenomas by snRNAseq. Larger-scale snRNAseq studies evaluating a broadly representative cohort of parathyroid adenomas could provide insight into how the potential suppressive mechanisms described here are altered in hypersecretory tumors. While the number of donor glands sequenced was limited, the number of nuclei evaluated and the read depth produced are both substantial and generated high confidence intervals and statistical significance in visualizing the discrete cellular populations within the normal gland. The unexpected availability of tumor-adjacent normal glands from a PHPT donor provided a unique opportunity to discern the response of normal tissue to a PTH hypersecretory environment, and confirmation of the respective transcriptional signatures in an independent set of normal donor tissues reinforced the conclusions drawn from primary sequencing dataset. Although we detected transcriptionally distinct subtypes, no structural or localization differences were observed, raising the possibility that these signatures represent different functional states of a common cell type rather than distinct, differentiated cellular identities. This outcome could indicate a greater degree of biochemical plasticity within the parathyroid gland than is currently appreciated and could suggest that the secretory activity of normal tissue may be therapeutically modulated using endogenous signaling pathways not solely dependent upon CASR. The increase in PTH secretion upon interference with LRP2 or PDE11A in live human parathyroid tissue suggests that these pathways may mediate physiological suppression and could presage a targetable approach for therapeutically inducing suppression in hypersecretory parathyroid glands.

## Data Availability

All original data generated and analyzed in this study are included in this published article or are contained in the data repositories listed in the references (https://doi.org/10.62251/fa:ds:TDLFBRA; https://doi.org/10.6084/m9.figshare.32209641, https://doi.org/10.6084/m9.figshare.30698369.v1).
